# A Low-Cost Porous Polymer Membrane for Gas Permeation

**DOI:** 10.3390/ma15103537

**Published:** 2022-05-14

**Authors:** Selim Haouari, Denis Rodrigue

**Affiliations:** Department of Chemical Engineering, Laval University, 1065 Avenue de la Médecine, Quebec City, QC G1V 0A6, Canada; selim.haouari.1@ulaval.ca

**Keywords:** LDPE, corn starch, porous membrane, leaching, gas separation

## Abstract

In this work, an efficient technique was used to produce porous membranes for different applications. Polyethylene (PE) was selected for the matrix, while corn starch (CS) was used to create the porous structure via leaching. The membranes were produced by continuous extrusion (blending)–calendering (forming) followed by CS leaching in a 20% aqueous acetic acid solution at 80 °C. A complete characterization of the resulting membranes was performed including morphological and mechanical properties. After process optimization, the gas transport properties through the membranes were determined on the basis of pure gas permeation including CH_4_, CO_2_, O_2_, and N_2_ for two specific applications: biogas sweetening (CH_4_/CO_2_) and oxygen-enriched air (O_2_/N_2_). The gas separation results for ideal permeability and selectivity at 25 °C and 1.17 bar (17 psi) show that these membranes are a good starting point for industrial applications since they are low-cost, easy to produce, and can be further optimized.

## 1. Introduction

The membrane gas separation industry has increased in size over the past four decades. This growth is related to the rapid development of more efficient membranes with higher perm-selectivity properties. The first industrial membrane for gas separation systems was tested in 1979 for the separation of H_2_ from N_2_, as well as CH_4_ and Ar [1]. In addition, the increasing demand for biogas production/purification, lower costs, and more efficient membrane separation are the main factors driving this market growth, especially for polymer-based membranes. Membranes for gas separation are also used for other applications, such as nitrogen generation and oxygen enrichment, hydrogen recovery, steam/gas separation, steam/steam separation, and air dehydration [2]. Today, a significant amount of work is dedicated to the development of polymeric membranes for gas separation, which is related to the numerous advantages of these membranes, such as low cost, low weight, and high efficiency, as well as easy production and simple operation/maintenance [3]. Today, membrane-based processes, especially porous polymeric films, receive a great deal of attention due to their simple use in separation and purification [4], and as solid supports for sensors and catalysts [5].

The membranes for gas separation can be classified into compact or porous structures. For the latter, specific morphological properties, such as pore size, shape, density, and internal surface area-to-volume ratio must be optimized to control the gas transport properties (diffusivity, permeability, selectivity, etc.), which are important for each membrane with respect to the gases (solubility) and separation conditions (composition, pressure, temperature, etc.). Although porous materials have been used, including mesoporous silica and alumina, polymers are of high interest because they are more ductile (less fragile) over a wide range of conditions (temperature) depending on the final application, e.g., gas separation, air/water filtration, and biological/biomedical purification [6].

Some studies reported on different methods to produce porous polymer membranes. For example, porous polyethylene membranes were successfully prepared from low-density polyethylene (LDPE)/tapioca starch using acidic and enzymatic leaching techniques. It was found that the formation of the porous structure was directly related to the amount of starch removed from the blends, as starch particles were most effectively removed by an aqueous solution of 5 N HNO_3_ at 65 °C, leading to a removal efficiency of 85% [7]. In another case, PE microporous membranes were also prepared using ultrahigh-molecular-weight polyethylene (UHMWPE) and liquid paraffin via thermally induced phase separation (TIPS). The quenching temperature and annealing time were the main parameters controlling the final porous structure with pore sizes of 3–5 μm and porosity of 50–60% [8]. Lastly, several works used salt leaching to generate the porosity. For example, highly hydrophobic microporous LDPE hollow-fiber membranes used for CO_2_ capture in gas–liquid membrane contactors were prepared using melt extrusion of LDPE/NaCl blends followed by salt leaching via immersion in water at 60 °C for 160 min. The membrane porosity reached 51% using 60–68 wt.% of salt with a pore diameter size in the range of 2–5 μm [9]. In another study, a simple and efficient method was used to produce a microporous LDPE support for a polydimethylsiloxane (PDMS) active layer by continuous extrusion and salt leaching (68 wt.% NaCl) via immersion in water at 50 °C. The results showed a pore size in the range of 8–11 μm, and the porosity was calculated by the volume of salt removed from the polymer (47%) [10]. Recently, scaffolds based on polylactic acid (PLA) reinforced with cellulose nanofibers (CNF) and salt were immersed in deionized water for 3 days to leach out the salt (porogen). The samples had interconnected pores with an average pore size in the range of 67–137 µm and porosities above 76% using solvent casting and particle leaching techniques [11].

Considering the information available in the literature, the main objective of this work was to produce low-cost porous polymer membranes with an easy-to-control simple process based on different formulations (concentration/composition) and extrusion parameters (temperature profile, screw speed, flow rate, etc.). To have a more sustainable product, a biobased material (corn starch) was combined with an easy-to-recycle polymer (polyethylene).

## 2. Experimental

### 2.1. Materials

Low-density polyethylene (LDPE) Novapol LA 0219-A (Nova Chemicals, Calgary, AB, Canada) with a density of 931 kg/m^3^ (ASTM D792) and a melt flow index of 2.3 g/10 min (ASTM D1238) was selected as the matrix, while a commercial (Great value) corn starch (CS) was used for the leaching phase. The leaching phase was performed using glacial acetic acid (99.7%) purchased from Anachemia (Lachine, QC, Canada).

### 2.2. Membrane Preparation

#### 2.2.1. Production of the Membrane by Extrusion

**Figure 1:**
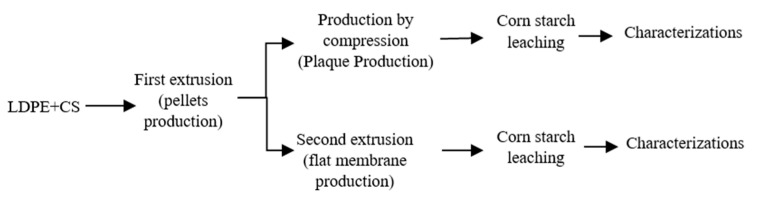
Schematic diagram of the membrane preparation and characterization steps.

As described in Figure 1, the first step (compounding) combined the LDPE (powder of 500 μm produced via a Powder King lab scale pulverizer, Anthem, AZ, USA) and corn starch with an average particle size of 10 μm. The compounds were prepared in a twin-screw extruder (Haake rheomex OS PTW16/40, De Soto, MO, USA) using a flat temperature profile of 105 °C, a screw speed of 60 rpm, and a feed rate of 3.8 g/min to obtain a homogeneous LDPE/CS blend. According to a series of preliminary experiments, the best LDPE/CS ratio was found to be 50/50 [12], because lower starch content did not produce porous structures after leaching, while higher CS content led to agglomeration (large pores) and low mechanical strength. The extrudate (circular die of 2 mm) was cooled down in a water bath at room temperature and pulled through a calender at a speed of 30 rpm. The compounds were then pelletized (Berlyn, PELL 2) and dried in an oven at 50 °C for 24 h. For the second extrusion step (flat membrane production), the LDPE/CS pellets were fed at a rate of 7 g/min in the same twin-screw extruder (Haake rheomex OS PTW16) by replacing the circular die by a flat one (50 mm × 1.7 mm). The die temperature was fixed at 130 °C with a screw speed of 50 rpm and a stretching speed (calender) of 5 rpm.

#### 2.2.2. Production of the Membrane by Compression

To account for the processing method (continuous vs. batch), some samples were also produced via compression molding (Figure 1). The pellets obtained from the first extrusion step (compounding) were placed in a square mold (10 cm × 10 cm × 0.8 mm). Both plates of the press were fixed at 130 °C. A preheating time of 3 min was applied before setting the force at 2 t for 5 min. Then, heating was stopped and cooling down to 30 °C was achieved via water circulation while keeping the pressure.

#### 2.2.3. Formation of the Porous Structure

**Figure 2:**
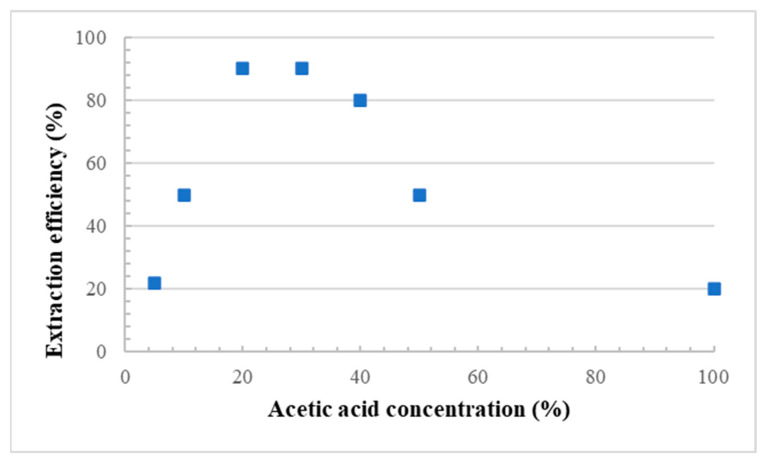
Corn starch extraction efficiency as a function of acetic acid concentration.

For each membrane, the porous structure was generated by removal (leaching) of the starch particles. Several conditions were investigated (Figure 2), but the optimum CS extraction was obtained using a 20% aqueous solution of acetic acid at 80 °C [12], while the leaching time was fixed at 48 h under continuous stirring of the liquid phase. To confirm the effect of acetic acid on particle leaching, a neat LDPE film (0 wt.% CS) was also immersed in the acid solution, and no weight loss was observed for the conditions tested. This indicates that all the weight loss observed can be associated with the hydrolytic removal of starch particles [7]. The amount of extracted starch (leaching efficiency) was calculated as follows [7]:

(1) $$\%\;\mathrm{extraction}=\frac{\mathrm{initial}\;\mathrm{mass}-\mathrm{final}\;\mathrm{mass}}{\mathrm{initial}\;\mathrm{mass}}\times \frac{100}{\%}\mathrm{initial}\;\mathrm{corn}\;\mathrm{starch}.$$

### 2.3. Characterizations

#### 2.3.1. Scanning Electron Microscopy (SEM)

The membrane morphology was analyzed by SEM using a JEOL JSM-840A (JEOL, Tokyo, Japan). The samples were first immersed in liquid nitrogen (30 s) and then broken to obtain cross-sections and longitudinal sections. The pore size distributions, pore density, and total porosity were calculated by analyzing the SEM images using the Image J (1.53e) software (National Institutes of Health, Bethesda, MD, USA). The pore diameter was determined by using the equivalent spherical area, while the pore density (*N*) was calculated as follows [13]:

(2) $$N=\left(N_{1}\right){\left(N_{2}\right)}^{\frac{1}{2}},$$

where *N*_1_ and *N*_2_ are the surface pore density in the longitudinal and transversal directions, respectively.

#### 2.3.2. Differential Scanning Calorimetry (DSC)

The thermal properties were determined in terms of crystallinity, melting temperature, and crystallization temperature by DSC (DSC 7, Perkin Elmer, Waltham, MA, USA). The measurements were performed using 5–10 mg of sample placed in an aluminum pan under a nitrogen atmosphere (20 mL/min). The temperature rate was set at 10 °C/min for a heating cycle from 50 to 200 °C, then a cooling cycle from 200 to 50 °C, followed by another heating cycle from 50 to 200 °C. The crystallinity was calculated as follows:

(3) $$X_{c}=\frac{\Delta H_{exp}}{\Delta H^{*}}\times 100,$$

where Δ*H_exp_* is the experimental enthalpy of fusion, and Δ*H* * is the enthalpy of fusion of the 100% crystalline polymer taken as 279 J/g for LDPE [14].

#### 2.3.3. Thermogravimetric Analysis (TGA)

TGA was used to confirm the mass loss of each sample (CS content) via thermal degradation. The samples were heated at a constant rate of 10 °C/min under air (thermo-oxidative decomposition) or N_2_ nitrogen (thermal decomposition) under a gas flow rate of 25 mL/min over a temperature range between 30 and 850 °C.

#### 2.3.4. Density

A Quantachrome (Boynton Beach, FL, USA) Ultrapyc 1200e gas (nitrogen) pycnometer was used to determine the density of the materials (CS, LDPE and blends). The reported values are the average of a minimum of three measurements.

#### 2.3.5. Tensile Properties

Tensile properties were characterized using an Instron universal testing machine (model 5565, Instron, Norwood, MA, USA) following ASTM D882. The dimensions were fixed at length = 9.53 mm and width = 3 mm, but the thickness varied between 0.9 and 1.1 mm as a function of the processing method and leaching efficiency. A load cell of 500 N and a crosshead speed of 10 mm/min at room temperature were used to report the results on the basis of a minimum of five repetitions.

#### 2.3.6. Contact Angle

Contact-angle measurements were performed with an optical contact angle (OCA 15 Plus) using the sessile drop method. A small water droplet was placed on the surface of a membrane, and the contact angles were determined from images acquired by an optical camera. At least three droplets were used on each membrane to get an average value which was associated with the level of hydrophobicity/hydrophilicity of the membrane surfaces.

#### 2.3.7. Gas Permeability

Gas permeation analyses were performed at 25 °C using a homemade setup according to the constant volume/variable pressure method [15]. The membranes were cut into discs of 50 mm in diameter with thicknesses around 1 mm. Each gas, N_2_, CO_2_, CH_4_, and O_2_ (Praxair, Quebec, QC, Canada), was charged to the feed side at a pressure of 17 psi, and the permeate side pressure (after vacuum) was measured as a function of time. The permeability *P* (Barrer) was determined as follows [16]:

(4) $$P=22,414\times 10^{10}\frac{V\;l}{R\;T\;A\;\left(p_{1}-p_{2}\right)}\left(\frac{dp}{dt}\right),$$

where *V* is the volume of the downstream chamber, *T* is the absolute temperature, *R* is the universal gas constant, *A* is the membrane area, *l* is the membrane thickness, *p*_1_ is the feed pressure, *p*_2_ is the permeate pressure, and ($\frac{dp}{dt}$) represents the slope of the permeability vs. time curve.

## 3. Results and Discussion

### 3.1. Morphology

#### 3.1.1. Starch Morphology

**Figure 3:**
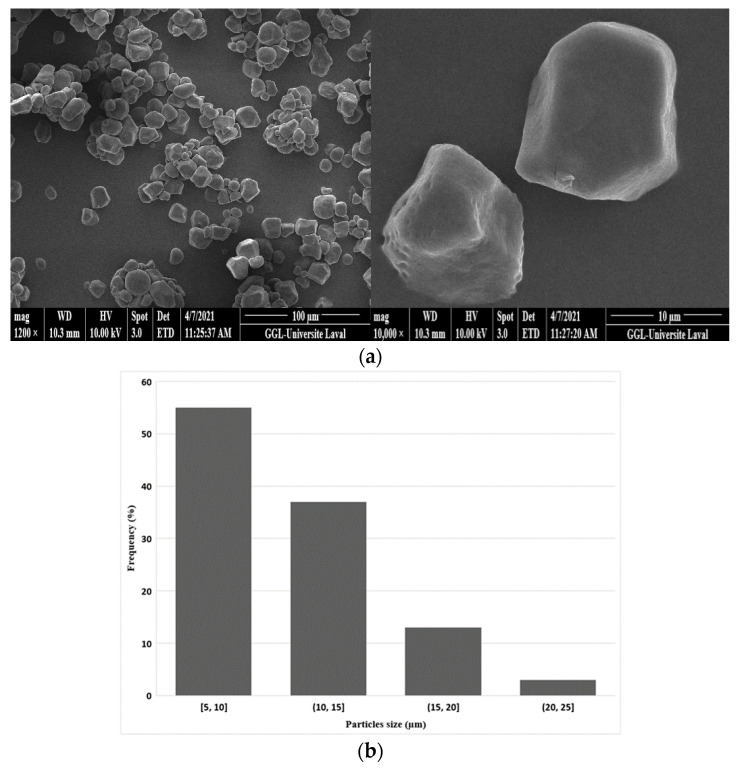
(**a**) Typical SEM images of the original corn starch particles and (**b**) their particle size distribution.

Figure 3a shows that the corn starch particles had a polyhedral shape and an alternating structure between amorphous and semicrystalline phases that were insoluble in water at room temperature. The crystallinity of starches is related to the double helical chains of amylopectin, the affinity of the crystalline zones, and intermolecular hydrogen bonds [17]. Analysis of the particle size distribution (Figure 3b) revealed an average size around 10 μm with a standard deviation of 4 μm.

#### 3.1.2. Morphology of LDPE Membrane after Leaching (Extrusion)

**Figure 4:**
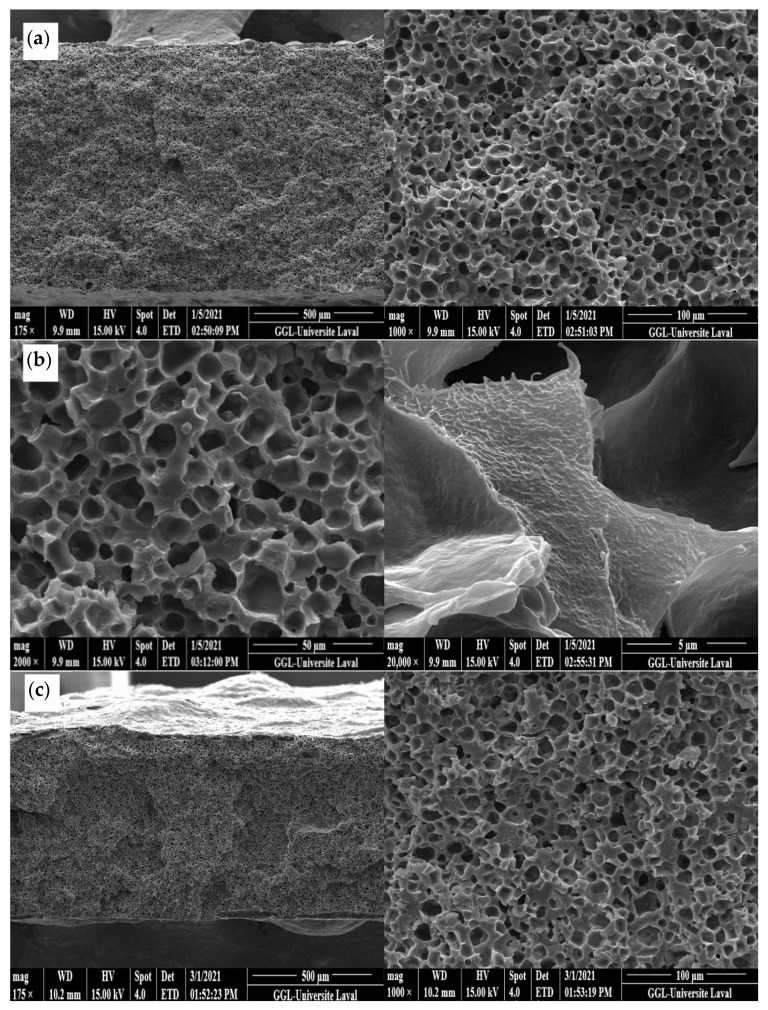

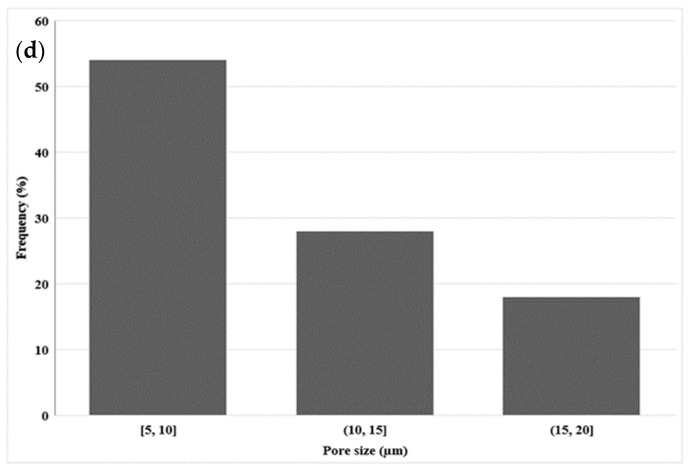
SEM images of: (**a**,**b**) the cross-section of the LDPE membrane (extrusion) at different magnification, (**c**) longitudinal section of the LDPE membrane (extrusion), and (**d**) pore size distribution inside the LDPE membrane.

Figure 4a presents SEM images of the LDPE membrane after CS leaching. It can be seen that the membrane had a microporous structure with a density of 811 pores/μm^3^ as calculated using Equation (2) and a CS extraction efficiency of 91% as calculated using Equation (1). As Figure 4b shows, there were still CS particles that were not extracted, confirming that 100% extraction was not achieved. Nevertheless, mainly open pores were observed allowing good connectivity, but some closed pores were visible, explaining why some CS particles were not completely extracted, as they were totally isolated (encapsulated) by the matrix. The pore size distribution inside the LDPE (Figure 4d) led to an average of 10 µm with a standard deviation of 5 µm. This shows that, during extrusion, the shear and elongation stresses did not modify the CS particles as the pore sizes were similar to the initial CS size (Figure 3).

#### 3.1.3. Morphology of LDPE Membrane after Leaching (Compression)

**Figure 5:**
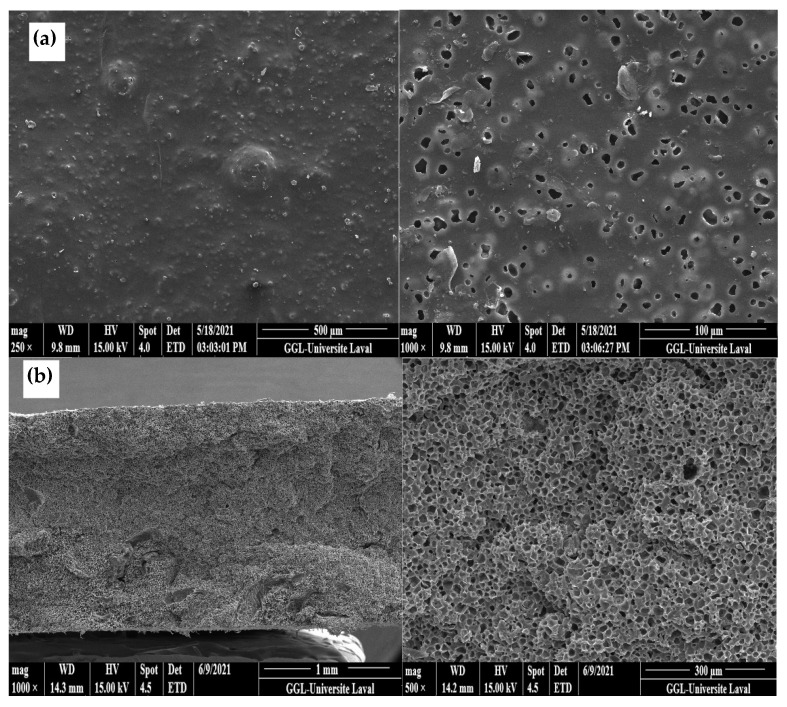

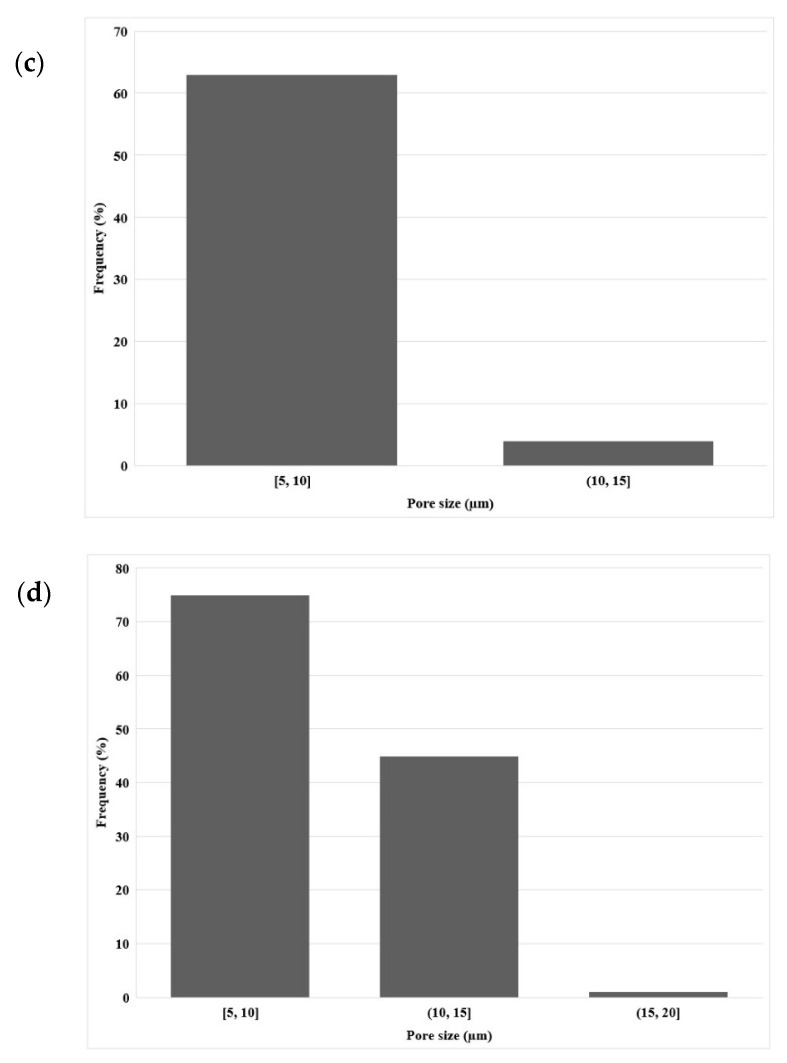
SEM images of: (**a**) LDPE membrane surface and (**b**) cross-section of the LDPE membrane after leaching (compression), (**c**) pore size distribution on the LDPE membrane surface, and (**d**) pore size distribution inside the LDPE membrane after leaching.

Figure 5a shows the presence of open pores on the membrane surface (7 µm with a standard deviation of 3 µm), which is similar in size to the pore size distribution inside the LDPE membrane (Figure 5b)m which had an average of 10 µm with a standard deviation of 3 µm. Again, the results are similar to the extruded LDPE microporous membrane (Figure 4) and the initial particle sizes (Figure 3). However, less agglomeration was seemingly present as large cavities could not be seen, leading in this case to a slightly higher extraction efficiency of 94%.

### 3.2. Differential Scanning Calorimetry

#### 3.2.1. Thermal Properties of Corn Starch

**Figure 6:**
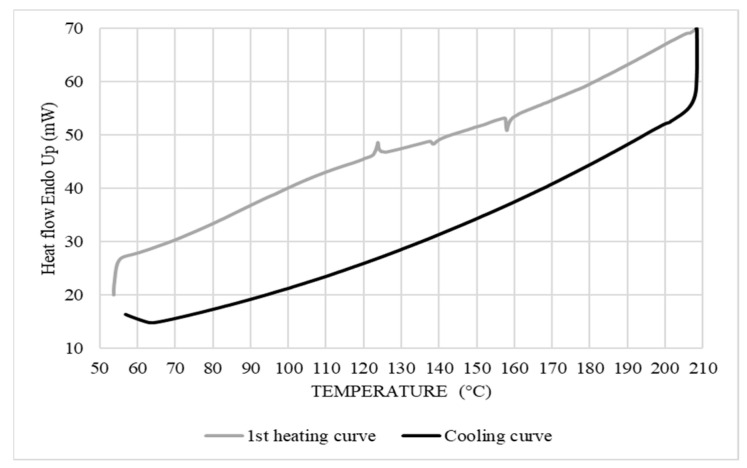
DSC curves of the corn starch (10 °C/min).

The melting temperature (*T_f_*) and enthalpy of melting (Δ*H_f_*) were determined from the endothermic melting curve, while the crystallization temperature (*T_c_*) and enthalpy of crystallization (Δ*H_c_*) were obtained during the cooling cycle (Figure 6). Corn starch has amorphous regions, crystalline regions, and lipid complexes of amylose with low water content, generating a high gelatinization temperature of 124 °C and Δ*H_f_* of 1.5 J/g. Thus, all these peaks could be observed, although the first peak was not very sharp with a *T_c_* of 138 °C, reflecting partial crystallization, while the presence of the crystallization peak at 158 °C with a Δ*H_c_* of 1.72 J/g upon heating was attributed to CS being a semi-crystalline polymer that can also undergo reorganization during the heating cycle [18].

#### 3.2.2. Thermal Properties of LDPE

**Figure 7:**
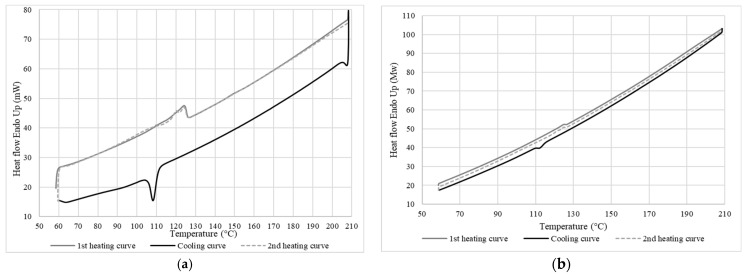
DSC curves of LDPE at a rate of (**a**) 10 °C/min (top) and (**b**) 2 °C/min (bottom).

The heating and cooling thermograms obtained by DSC for LDPE are presented in Figure 7, while Table 1 reports the values for the melting temperature (*T_f_*), crystallization temperature (*T_c_*), enthalpy of melting (Δ*H_f_*), enthalpy of crystallization (Δ*H_c_*), and degree of crystallinity (*X_c_*) for this LDPE. The *T_f_* of 125 °C is very high compared to the expected melting temperature for pure LDPE which is usually around 114 °C [19,20]. After erasing the thermal history, the second heating curve showed the presence of two nonseparated melting peaks with a maximum peak of 124 °C and a second one at 120 °C to give Δ*H_f_* = 18 J/g. A second DSC analysis was performed at a lower heating rate (2 °C/min) to verify this bimodal behavior. However, Figure 7 shows the same result as for a heating rate of 10 °C/min. This can indicate two things: either the LDPE had some contamination/additive (a copolymer or a mixture of different types of PE), or that a single material was present but with two different crystal structures or a wide distribution of molecular weight [21]. However, the cooling curve showed a single crystallization peak. Therefore, it the second case is most likely, i.e., a single material with two peaks, corresponding to a semicrystalline structure peak (high peak) and a high-energy crystalline structure peak which can melt more easily (low peak).

#### 3.2.3. Thermal Properties of the Membrane

**Figure 8:**
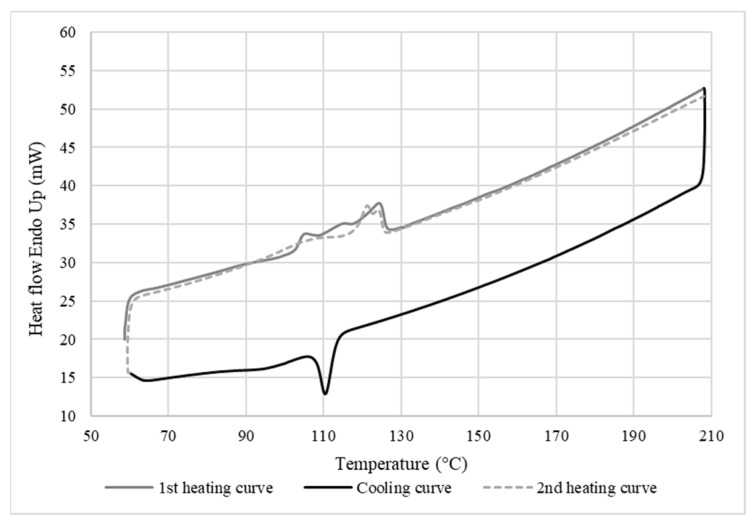
DSC curves of the membrane after leaching (10 °C/min).

Figure 8 shows that the melting and crystallization temperatures of the LDPE membrane after leaching were the same as the neat LDPE (Figure 7), indicating that the LDPE/CS blends were incompatible. Furthermore, no significant increase in membrane crystallinity was observed (*X_c_* = 17%), which supports the SEM analyses (Figure 4), indicating that CS was not fully extracted and that both phases were immiscible or had very weak interactions. The triple peaks at 104 °C, 114 °C, and 124 °C obtained during the melting of LDPE show that the matrix was composed of crystals of different sizes and types (different types of nucleation), indicating that the low number of small CS particles remaining could act as heterogeneous nucleation sites.

### 3.3. Thermogravimetric Analysis

#### Thermal Stability of the LDPE Membrane

**Figure 9:**
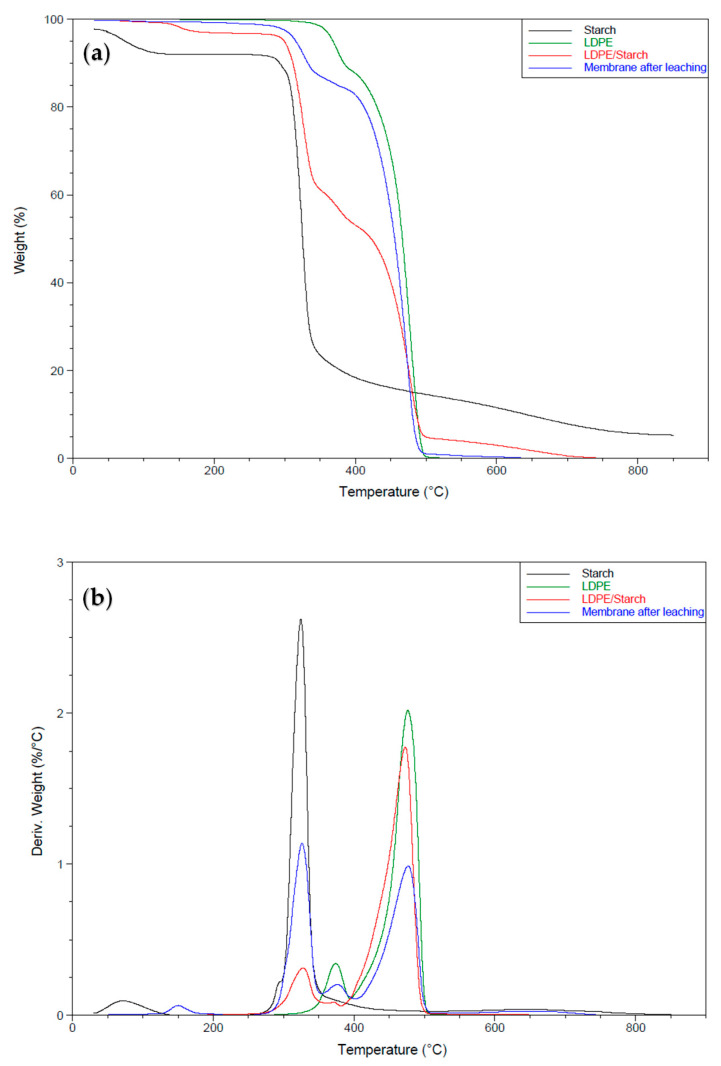
(**a**) Thermogravimetric analysis curves of LDPE, CS, and the membrane under N_2_ and (**b**) first derivative of the curves presented in (**a**).

Figure 9 presents the thermograms under N_2_, while Table 2 summarizes the TGA results of the curves for CS, LDPE, and the membrane. The results show two distinct weight losses for the CS curve. A first loss (5.7%) with an initial decomposition temperature between 40 and 135 °C corresponded to the loss of volatile products (mainly water) [22], while the second was associated with the decomposition of the starch molecules between 253 °C and 650 °C (82.2% loss) [23]. Above this temperature, the weight loss (4.4%) could be associated with inorganic impurities and residual carbon [24]. The neat LDPE curve also showed two weight losses: the first loss (11%) with a decomposition temperature between 317 °C and 392 °C corresponding to a compound with a low molecular weight, and a second loss (88%) between 393 and 500 °C corresponding to the main LDPE chain [25]. This indicates that only one material (LDPE) was present, but with a different morphology (nucleation), as reported by the DSC results (Figure 7). The curve for the LDPE/CS sample showed four mass losses. The first (2.7% between 130 °C and 190 °C) was associated with the loss of small molecules (volatiles) not removed during extrusion. The second loss (34.3% between 278 °C and 344 °C) was associated with the thermal degradation of CS. However, this indicates that the thermal stability of CS decreased during blending (Table 2), while the thermal degradation of LDPE remained similar to the neat LDPE with two successive losses of 8% and 49% in the ranges 344–391 °C and 391–500 °C, respectively. The residue (4.9%) at 500 °C was considerably higher than for the neat LDPE, which was associated with CS. This curve also shows that the mass loss associated with CS (41%) was slightly lower than its content in the initial mixture (LDPE/CS = 50/50). This means that the distribution of CS in the matrix was not totally uniform or that the extraction rate was not uniform (or a combination of both effects). The curve for the LDPE membrane after extraction showed three mass losses. The first loss (2.4% between 178 °C and 303 °C) was negligible and associated with the thermal degradation of residual CS, confirming the results of a high extraction rate. The second loss (13% between 303 °C and 389 °C) corresponded to additives, while the third loss (83% between 389 °C and 496 °C) was associated with the complete thermal degradation of LDPE. For the conditions used, the thermal stability of LDPE in the membrane was similar to the neat LDPE, i.e., no change was associated with the blends with CS in the extruder.

### 3.4. Density

Table 3 summarizes the density obtained for the membranes before and after starch extraction, compared to that of the base materials. It can be seen that adding 50% CS increased the matrix density by 20% (0.932 g/cm^3^ to 1.125 g/cm^3^), associated with the higher density of CS (1.504 g/cm^3^) compared to LDPE (0.932 g/cm^3^) and the interactions between the phases. In fact, LDPE is nonpolar (hydrophobic), while CS is a polar (hydrophilic) compound, which can lead to voids at their interfaces (incompatibility). However, for this application, poor interfacial adhesion was useful to create paths for the leaching solution and to maximize the extraction process and/or the passage of gas molecules [26]. As expected, the density decreased by 19% (1.125 g/cm^3^ to 0.915 g/cm^3^) after CS extraction by creating a porous structure in the matrix, as shown in the SEM images (Figure 4 and Figure 5).

### 3.5. Tensile Properties

#### Mechanical Properties of the LDPE Membrane

**Figure 10:**
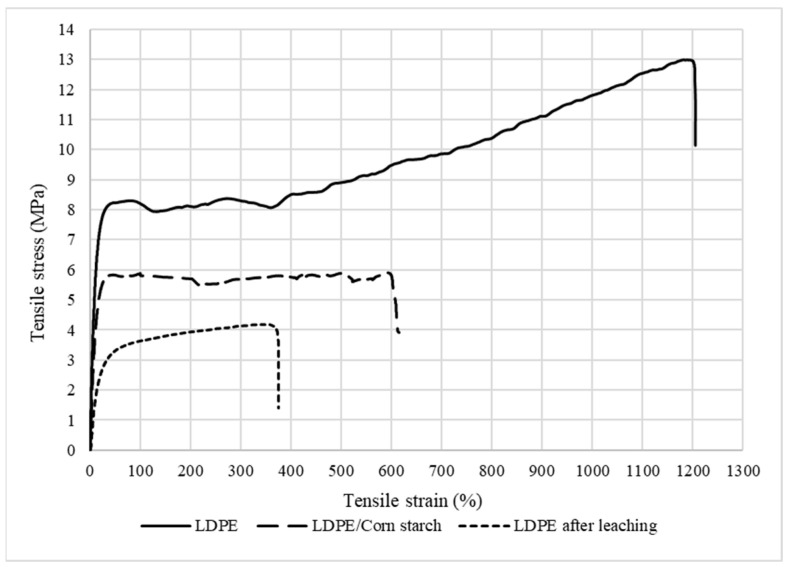
Tensile stress–strain curves for the different samples.

The tensile mechanical properties are summarized in Table 4 according to the stress–strain curves presented in Figure 10. It can be noted that the addition of CS decreased the Young’s modulus of LDPE from 84 to 68 MPa, as well as the tensile strength from 8 to 5 MPa. These decreases were associated with a lack of compatibility between both components due to their different hydrophilic/hydrophobic characters (corn starch vs. LDPE). In addition, the lower LDPE tensile strength after CS addition is again an indication of weak interfacial interactions between the components, leading to mechanical failure at their interface [27]. Another explanation is linked to the intrinsic properties of starch. It is known that corn starch is brittle and has low mechanical properties, such as tensile strength (1.49 MPa), elongation at break (51%), and Young’s modulus (14.2 MPa) [28]. Lastly, the membrane after extraction had the lowest tensile properties due to the formation of the porous structures (presence of voids in Figure 4 and Figure 5). In this case, there was less material available to sustain the applied stresses.

### 3.6. Contact Angle

The contact angles are summarized in Table 5. The values provide information on the degree of hydrophobicity/hydrophilicity of the membrane surfaces. The average contact angle was found to be 96° for the porous LDPE membrane. These results indicate the formation of more hydrophobic surfaces of LDPE and that most of the CS was extracted since CS is a strongly hydrophilic compound (low contact angle value), because higher membrane surface hydrophobicity (high contact angle) leads to lower swelling effects related to possible humidity in the feed gas, thus decreasing the transport of molecules through the membrane [29]. In addition, the results show a slight difference between the top and bottom faces. This could be related to different surface roughness of the calendar rolls used (wear).

### 3.7. Permeability

Table 6 presents the results of gas permeation through the porous LDPE membrane (compression) under a pressure of 17 psi at 25 °C. The permeability increased in the following order: O_2_ < N_2_ < CO_2_ < CH_4_, while the kinetic diameter of the tested gases increased in the following order: CO_2_ < O_2_ < N_2_ < CH_4_. The gas solubility and affinity toward the remaining CS inside and/or between the pores (Figure 4 and Figure 5) could have influenced this difference in order.

Neat LDPE is known to have low gas permeability because the matrix is compact (low free volume) and has some crystalline zones acting as gas barriers, thus increasing the tortuosity and mean free path of the penetrating gas molecules [30]. This is also related to the mobility restriction of the polymer chains by the crystallites [31]. By adding CS to the matrix, the permeability increased due to the creation of free volume/porosity between the chains/phases because of the poor compatibility between both materials and lower crystallinity (Table 1). However, after CS extraction, the final structure was a combination of some closed pores with a network of interconnected pores, leading to higher transport properties (Figure 4 and Figure 5). In polymer membranes, the solution–diffusion theory applies, but each process is controlled by the gas molecules size and the membrane’s pore sizes. Thus, the creation of the porous structure increased the gas permeability leading to much higher values, but selectivity is the main parameter for separation applications. In our case, the values were not improved, indicating that permeability improvement was similar for all the gases (similar diffusion velocity) through the membrane. This can be improved by adding functional (nano)particles, such as zeolites or trimethylsiloxy grafted fumed silica (TFS), as well as by adding a polydimethylsiloxane (PDMS) active layer to coat a porous support membrane to produce mixed matrix membranes (MMM) [32,33]. These improvements will be the subject of a future study.

Except for CO_2_/N_2_, Table 6 shows that the ideal selectivities for the selected gases were above 1.5, indicating that gas separation was possible. The best result (α = 3.1) was obtained for the CH_4_/O_2_ system, which could be of interest to remove oxygen traces in biogas production (methane). Although these selectivities were not very high (1.6–3.1), the permeabilities were very impressive (10^4^–10^5^ Barrer). 

**Figure 11:**
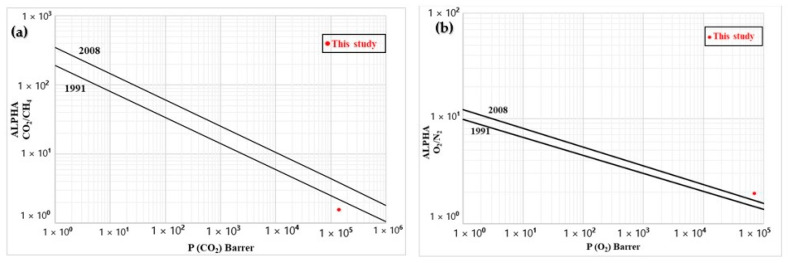

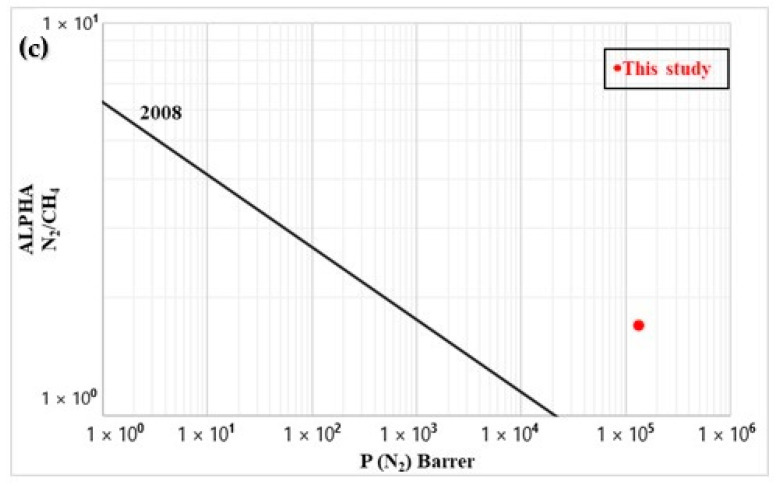
Comparison between the experimental data and Robeson upper bounds for (**a**) CO_2_/CH_4_, (**b**) O_2_/N_2_, and (**c**) N_2_/CH_4_.

As a tradeoff always occurs between selectivity (α) and permeability (*P*), Figure 11 compares the results with standard Robeson upper bounds [34,35]. The CO_2_/CH_4_ (biogas sweetening) data were close to the 1991 upper bounds, while they overcame the 2008 bound for O_2_/N_2_ (oxygen-enriched air). For N_2_/CH_4_ (natural gas purification), the data were well above the 2008 upper bound. These results indicates that the membrane prepared can be a very good starting point for specific high-throughput separation of these gases (CO_2_/CH_4_, O_2_/N_2_, and N_2_/CH_4_), which will be the focus of future work.

## 4. Conclusions

In this work, a simple methodology was presented to produce a flat porous polymer membrane without chemical agents or toxic solvents. As a first step, virgin LDPE was used as the matrix with corn starch as a biobased leachable particle. Nevertheless, to reduce the production cost and keep the sustainability of the process, recycled LDPE (or any other thermoplastic resin) and/or other soluble biobased particles can be used.

The process is a continuous extrusion–calendering blending/forming step coupled with an immersion leaching step to create a porous structure. The latter can be optimized/controlled by a careful selection of the particle size and concentration. In our case, a 10 μm commercial corn starch was used at 50 wt.% as a proof of concept. After optimization, an aqueous solution of diluted acetic acid (20%) was selected to remove the CS with high efficiencies (over 91%), which was confirmed via TGA. Overall, the proposed methodology is simple and economical since it is based on low-cost materials and can be continuously operated. It was also shown via DSC that the CS particles can improve the LDPE crystallinity, leading to a more tortuous path for the gas molecules. 

Lastly, the permeation results for CO_2_, CH_4_, O_2_, and N_2_ were measured under standard conditions (25 °C and 17 psi). Although the ideal selectivities were not very high (1.6–3.1), the permeability was impressive (7 × 10^4^–2 × 10^5^ Barrer) leading to conditions above or very close to the 2008 Robeson upper bounds.

With these properties, it is expected that these membranes could achieve reasonable separation performance, at least at a laboratory scale for applications such as biogas sweetening (CO_2_/CH_4_), oxygen-enriched air (O_2_/N_2_), and natural gas purification (N_2_/CH_4_). These membranes can be used alone in contactors with recycling streams to improve gas separation efficiency or serve as a porous support to coat with an active layer. There is also the possibility to produce hollow fibers using similar conditions by changing the die geometry. All these aspects will be investigated in a future study.

## Figures and Tables

**Table 1:** LDPE parameters obtained by DSC.

Matrix	*T_f_* (°C)	Δ*H_f_* (J/g)	*T_c_ *(°C)	Δ*H_c_* (J/g)	*X_c_* (%)
LDPE (10 °C/min)	125	23.0	108	28.0	8.2
LDPE (2 °C/min)	125	5.3	112	16.4	5.7

**Table 2:** TGA results for CS, LDPE and the membrane.

Curves	*T_d_*_1_ ^1^ (°C)	*T_d_*_2_ ^1^ (°C)	*T_d_*_3_ ^1^ (°C)	*T_d_*_4_ ^1^ (°C)	*W_d_*_1_ ^2^ (%)	*W_d_*_2_ ^2^ (%)	*W_d_*_3_ ^2^ (%)	*W_d_*_4_ ^2^ (%)	Residue (%)
CS	40–135	253–650	-	-	5.7	82.2	-	-	4.4
LDPE	317–392	393–500	-	-	11.0	88.0	-	-	0.5
LDPE/CS	130–190	278–344	344–391	391–500	2.7	34.3	8.0	49.0	4.9
Membrane after leaching	178–303	303–389	389–496	-	2.4	13.0	83	-	1.3

^1^ *T_d_*: decomposition temperature. ^2^ *W_d_*: weight loss.

**Table 3:** Density (*ρ*) of the different materials.

Materials	*ρ* (g/cm^3^)
CS	1.504
LDPE	0.932
LDPE/CS	1.125
Membrane after leaching	0.915

**Table 4:** Mechanical properties in tension: tensile stress at yield (*σ*_0_), strain at yield (*ε*_0_), tensile stress at break (*σ*_*max*_), strain at break (*ε*_*max*_), energy at break (*En*), and Young’s modulus (*E*).

	$\sigma_{0}$ (MPa)	$\varepsilon_{0}\;(\%)$	$\sigma_{max}\;(\mathrm{MPa})$	$\varepsilon_{max}\;(\%)$	En (J)	*E* (MPa)
LDPE	8.3 (±0.2)	79 (±5)	12.3 (±0.6)	1204 (±11)	3.03 (±0.27)	84.9 (±2.4)
LDPE/CS	5.8 (±1.0)	46 (±8)	4.6 (±2.0)	628 (±9)	1.28 (±0.49)	63.5 (±10.0)
LDPE after leaching	4.2 (±0.2)	334 (±9)	2.6 (±0.4)	375 (±9)	0.49 (±0.11)	21.1 (±1.1)

**Table 5:** Contact angle (°) measured at three different positions of the membranes after extraction.

Face	1	2	3	Standard Deviation
Top	95.6	95.8	98.1	1.0
Bottom	93.8	94.9	97.6	1.4

**Table 6:** Gas permeation results (CO_2_, CH_4_, O_2_, and N_2_) of the membrane.

Membrane	Permeability (10^5^ Barrer)				Ideal Selectivity (-)					
	CO_2_	CH_4_	O_2_	N_2_	CO_2_/N_2_	CO_2_/CH_4_	CO_2_/O_2_	CH_4_/O_2_	CH_4_/N_2_	O_2_/N_2_
LDPE after leaching	1.37	2.20	0.71	1.33	1.0	1.6	1.9	3.1	1.7	1.9

## Data Availability

The data presented in this study are available from http://hdl.handle.net/20.500.11794/72981, accessed on 11 May 2022.
